# Atorvastatin Upregulates microRNA-186 and Inhibits the TLR4-Mediated MAPKs/NF-κB Pathway to Relieve Steroid-Induced Avascular Necrosis of the Femoral Head

**DOI:** 10.3389/fphar.2021.583975

**Published:** 2021-04-16

**Authors:** Yusong Zhang, Limin Ma, Erhai Lu, Wenhua Huang

**Affiliations:** ^1^ Department of Orthopedics, Xinhui People’s Hospital of Southern Medical University, Jiangmen, China; ^2^ Guangdong Engineering Research Center for Translation of Medical 3D Printing Application, Guangdong Provincial Key Laboratory of Medical Biomechanics, National Key Discipline of Human Anatomy, School of Basic Medical Sciences, Southern Medical University, Guangzhou, China; ^3^ Guangdong Medical Innovation Platform for Translation of 3D Printing Application, Southern Medical University, The Third Affiliated Hospital of Southern Medical University, Southern Medical University, Guangzhou, China; ^4^ Orthopaedic Center, Affiliated Hospital of Guangdong Medical University, Guangdong Medical University, Zhanjiang, China

**Keywords:** steroid induced avascular necrosis of the femoral head, autophagy, atorvastatin, microRNA-186, TLR4, MAPKs/NF-κB pathway

## Abstract

Steroid-induced avascular necrosis of the femoral head (SANFH) is caused by the death of active components of the femoral head owing to hormone overdoses. The use of lipid-lowering drugs to prevent SANFH in animals inspired us to identify the mechanisms involving Atorvastatin (Ato) in SANFH. However, it is still not well understood how and to what extent Ato affects SANFH. This study aimed to figure out the efficacy of Ato in SANFH and the underlying molecular mechanisms. After establishment of the SANFH model, histological evaluation, lipid metabolism, inflammatory cytokines, oxidative stress, apoptosis, and autophagy of the femoral head were evaluated. The differentially expressed microRNAs (miRs) after Ato treatment were screened out using microarray analysis. The downstream gene and pathway of miR-186 were predicted and their involvement in SANFH rats was analyzed. OB-6 cells were selected to simulate SANFH *in vitro*. Cell viability, cell damage, inflammation responses, apoptosis, and autophagy were assessed. Ato alleviated SANFH, inhibited apoptosis, and promoted autophagy. miR-186 was significantly upregulated after Ato treatment. miR-186 targeted TLR4 and inactivated the MAPKs/NF-κB pathway. Inhibition of miR-186 reversed the protection of Ato on SANFH rats, while inhibition of TLR4 restored the protective effect of Ato. Ato reduced apoptosis and promoted autophagy of OB-6 cells by upregulating miR-186 and inhibiting the TLR4/MAPKs/NF-κB pathway. In conclusion, Ato reduced apoptosis and promoted autophagy, thus alleviating SANFH via miR-186 and the TLR4-mediated MAPKs/NF-κB pathway.

## Introduction

Steroid-induced avascular necrosis of the femoral head is a frequently occurring form of non-traumatic osteonecrosis and is a progressive pathological process caused by high dose and/or long-term use of dexamethasone (DEX) or other glucocorticoids (Lin et al., 2019; Cui et al., 2020). The main features of SANFH are degeneration of the femoral head and necrosis of bone trabecula or bone marrow, widespread bone marrow oedema, and fat cell proliferation (Erken et al., 2012). The typical pathological changes of SANFH include decreased osteogenic differentiation, enhanced intramedullary adipocytes deposition, and impaired osseous circulation (Xu et al., 2020). Due to the difficulty of early diagnosis and relatively poor medical conditions in some areas, some patients may be misdiagnosed and delayed being cured, leaving a high disability rate (Li et al., 2019). Statistical investigations have demonstrated that, if not treated properly, more than 50% of SANFH cases could ultimately lead to bone fractures (Huang et al., 2019a). By virtue of the development of molecular biology, a strong association of apoptosis with the pathogenesis of SANFH has been inferred (Cui et al., 2020). Therefore, a comprehensive understanding of the apoptosis mechanisms of osteocytes will provide a novel insight to clarify the initiation and progression of SANFH, as well as to develop proper therapies to minimize or even eliminate the progression of it.

Osteoblasts play pivotal roles in regulating bone growth and bone matrix formation (Himburg et al., 2010; Lin et al., 2020). In recent years, it has been demonstrated that femoral head necrosis is closely related to apoptosis of mature osteoblasts and osteoclasts (Bai et al., 2015). Glucocorticoids may decrease osteogenesis and increase adipogenesis of the bone marrow during the process of SANFH (Feng et al., 2017). DEX can induce oxidative injury, apoptosis and programmed necrosis of primary osteoblasts (Liang et al., 2019; Zhu et al., 2019). The cultured osteoblasts added with DEX were used to create a cellular model of SANFH (Huang et al., 2019a; Lin et al., 2019). Despite the strong association between DEX usage and osteonecrosis, the exact mechanism of SANFH is unclear. A determination of the pathological mechanisms of the DEX-induced model will facilitate the development of pharmacological agents that block SANFH initiation and progression clinically.

It has been proven that the changes of systemic or local lipid metabolism may play an important role in SANFH, and the use of lipid-lowering drugs to prevent SANFH in animals further supports this theory (Erken et al., 2012). Atorvastatin decreases cholesterol biosynthesis by inhibiting the HMG-CoA reductase enzyme and shows lipid-lowering activity (Meor Anuar Shuhaili et al., 2017). The lipid-lowering quality of Ato encourages us to speculate that Ato can relieve SANFH-induced injury. A previous study indicated that femurs appear to be more affected by a combination of Ato calcium and corticosteroid treatment, while avascular necrosis of the femoral head is a well-known adverse effect of corticosteroid therapy (Handal et al., 2012). The combined action of Ato and noncoding RNAs has also been increasingly researched. An integrated analysis of noncoding RNA and mRNA profiles in oxidized low-density lipoprotein-induced endothelial dysfunction after Ato administration further evidences this synergistic effect (Jiang et al., 2018). Noncoding RNAs, including microRNAs (miRs), are potent regulators of gene expression and function at transcriptional and posttranscriptional levels in the pathogenesis of SANFH (Wu et al., 2019). In the preliminary experiments of this study, miR-186 was significantly upregulated after Ato treatment.

Toll-like receptor 4 (TLR4) takes part in the anti-apoptotic role of miR-186 in podocytes (Sha et al., 2015) and in the process of steroid-induced osteonecrosis (Tian et al., 2014). TLR4 is enhanced after cetuximab treatment, with activation of downstream nuclear factor-kappa B (NF-κB) and mitogen-activated protein kinases (MAPKs) pathways (Ju et al., 2020). Moreover, reduced NF-κB expression is accompanied with diminished bone resorption and apoptosis in SANFH (Pei et al., 2017). Importantly, Ato relieves the renal inflammatory response by blocking the TLR4/NF-κB axis (Sun et al., 2020). Beyond that, inhibiting caspsae-3-mediated apoptosis may provide an alternative treatment for SANFH (Cui et al., 2018). Autophagy also participates in the pathological process of SANFH and is closely related to apoptosis (Luo et al., 2018).

So far, the correlation of Ato, miR-186, and the TLR4/MAPKs/NF-κB axis with autophagy and apoptosis in SANFH remains to be studied. In the light of this information, we hypothesized that Ato upregulates miR-186 and inhibits the TLR4-mediated MAPKs/NF-κB pathway in SANFH. We conducted histochemical and molecular experiments to verify the hypothesis.

## Methods

### Ethics Statement

This study was ratified and supervised by the ethics committee of the National Key Discipline of Human Anatomy, School of Basic Medical Sciences, Southern Medical University (animal ethical protocol number: 2020-010). Significant efforts were made to minimize the animals used and their pain.

### Establishment of SANFH Rat Model

Adult male Sprague Dawley rats (*n* = 50, 250–280 g) (Xu et al., 2018; Xue et al., 2018) purchased from Southern Medical University (SCXK (Guangdong) 2016-0041) were raised at 24 ± 2°C with a free diet and drinking water and a 12 h light/dark cycle.

SANFH rat model (*n* = 44) was induced by 50 mg/kg DEX injected into the gluteus muscle twice a week for 6 weeks, and 36 rats with successful modeling were selected for the following experiments (Ye et al., 2019). The successful modeling rats were assigned into the SANFH group (modeling rats, *n* = 6), and the remaining rats were administrated with Ato (15 mg/kg/day), (Baranowski et al., 2019) for two weeks after DEX injection. The six SANFH rats only administrated with Ato were set as the SANFH + Ato group, while the remaining 24 rats were randomly allocated into the ant-NC group (injected with negative control antagomir via tail vein), the ant-miR-186 group (injected with miR-186 antagomir via tail vein), and the ant-miR-186 + DMSO group (injected with miR-186 antagomir via tail vein, and intraperitoneally injected with 1 mg/kg TAK242 (TLR4 pathway inhibitor) (Selleck Chemicals, Houston, TX, United States) (Zhong et al., 2019) 30 min before sampling]. miR-186 antagomir and NC antagomir were synthesized by GenePharma Co., Ltd. (Shanghai, China). The rats in the control group were injected with the same amount of normal saline as the model group instead of DEX, and the same amount of placebo was orally administered in the same way as Ato (Table 1 for details of grouping treatment, and Supplementary Figure S1 for experimental design scheme). After modeling, all rats were euthanized via an intraperitoneal injection of excess pentobarbital sodium. The femoral heads of one side were used for real-time quantitative polymerase chain reaction (RT-qPCR) and Western blot analysis, and the other side for histological evaluation.

**TABLE 1:** Details of rat grouping treatment.

Group (*n* = 6)	Treatment
Control	PBS was injected into gluteus muscle twice a week for 6 weeks, and placebo was taken 2 weeks after PBS injection
SANFH	DEX was injected into gluteus muscle twice a week for 6 weeks, and placebo was taken 2 weeks after DEX injection
SANFH + Ato	DEX was injected into gluteus muscle twice a week for 6 weeks, and Ato was taken 2 weeks after DEX injection
ant-NC	The treatment was the same as SANFH + Ato group, but NC antagomir was injected via tail vein 2 weeks after DEX injection
ant-miR-186	The treatment was the same as SANFH + Ato group, but miR-186 antagomir was injected via tail vein 2 weeks after DEX injection
ant-miR-186 + DMSO	The treatment was the same as that of ant-miR-186 group, and DMSO was injected intraperitoneally at 30 min before sampling
ant-miR-186 + TAK-242	The treatment method was the same as that of ant-miR-186 group, and 1 mg/kg TAK242 was intraperitoneally injected 30 min before sampling

### Histological Evaluation

The femoral head of one side was fixed with 4% (m/v) paraformaldehyde at 4°C for 24 h and transferred to 10% (m/v) ethylene diamine tetraacetic acid for decalcification. After that, the samples were dehydrated in gradient ethanol, embedded in paraffin, and cut into tissue sections at 4 μm. Then the sections were stained using hematoxylin and eosin (HE) and observed with an optical microscope (×200) (Olympus Optical Co., Ltd., Tokyo, Japan). Three researchers counted the ratio of empty lacunae in a double-blinded manner. The paraffin sections were stained with TUNEL kit (Roche Diagnostics, Mannheim, Germany) and visualized with 2,4-diaminobutyric acid. TUNEL-positive cells with brown nuclei were observed under an optic microscope (×200).

### Enzyme-Linked Immunosorbent Assay

Blood samples of experimental rats were collected under anesthesia and centrifuged at 12,000 xg at 4°C for 10 min. Serum osteocalcin (OST, H152), total cholesterol (TC, A111-1-1), and the ratio of low-density lipoprotein to high-density lipoprotein (LDL/HDL; A113-1-1 and A112-1-1) were determined using ELISA kits (Nanjing JianCheng Bioengineering Institute, Nanjing, Jiangsu, China).

The levels of inflammatory factors, oxidative stress, alkaline-phosphatase (ALP), OST, and caspase-3 activity were measured in the femoral heads of rats. RIPA buffer (Beyotime Biotechnology Co., Ltd., Shanghai, China) was used to split the sample for 1 h and samples were centrifuged at 4°C at 12,000 xg for 10 min to collect the supernatant. The inflammatory factors tumor necrosis factor (TNF)-α (H052) and interleukin (IL)-1β (H002), and the oxidative stress indices (malondialdehyde, MDA, A003-1–2; superoxide dismutase, SOD, A001-3-2), catalase (CAT, A007-1-1), ALP (A059-2-2) and OST (H152), and caspase-3 activity were detected using corresponding kits (Beyotime) (Xiao et al., 2018; Ye et al., 2019).

### Dual Luciferase Reporter Gene Assay

According to starbase (http://starbase.sysu.edu.cn/index.php) (Li et al., 2014), there are binding sites between miR-186 and TLR4. The specific binding fragment and its mutant (MUT) were cloned into pmiR-GLO dual luciferase vector (firefly and renilla) (Promega, Madison, WI, United States), and recorded as wild-type (WT)-TLR4 and MUT-TLR4. In accordance with the instructions, Lipofectamine^TM^ 2000 (Invitrogen Inc., Carlsbad, CA, United States) was utilized to co-transfect NC mimic and miR-186 mimic into 293T cells (China Center for Type Culture Collection, Wuhan, Hubei, China). Luciferase activity (firefly and renilla) was detected 48 h later using Dual-Luciferase Reporter Assay System (Promega).

### Cell Culture and Grouping

According to the previous study (Ding et al., 2015), OB-6 cells were purchased from the Cell Bank of CAS Shanghai Institute of Biological Sciences (Shanghai, China). DEX was used to pretreat cells for 24 h, and the effect of DEX at different concentrations (0.25, 0.5, 1, 2, 5 μM) was observed using 3-(4, 5-dimethylthiazol-2-yl)-2,5-diphenyltetrazolium bromide (MTT) assay, and the IC50 values were calculated. Then the cells pretreated with 1 μM DEX were treated with 0, 0.25, 0.1, 1, or 5 μM Ato for 72 h (Yan et al., 2015). In addition, at the same time of 0.1 μM Ato treatment, cells were transfected with miR-186 inhibitor or the NC (GenePharma) according to Lipfectamine^TM^ 2000.

According to the previous studies (Muller et al., 2017; Pourgonabadi et al., 2017), miR-186 inhibitor-treated cells were transfected with 25 μM TAK242 or dimethyl sulfoxide (DMSO) (Beijing Solarbio Science & Technology Co., Ltd., Beijing, China) for 72 h for subsequent experiments.

### Monodansylcadaverine Staining

According to the previous studies (Wang et al., 2019b; Wang et al., 2019c), cells in each group were stained using the MDC kit (Solarbio, G0170) and observed under the fluorescence microscope (×400).

### MTT Assay

Cells were subcultured into 96-well plates at 2 × 10^3^ cells/well and cultured overnight. Then cells were cultured with fresh medium containing 20 μL 5 mg/ml MTT solution (Solarbio) for 4 h. Subsequently, the medium and MTT were removed. MTT formazan was extracted with DMSO and the absorbance value at 570 nm was determined. Six replicate wells were set for each treatment to get the average value.

### Detection of Lactate Dehydrogenase Release

LDH released by cells in culture medium was analyzed by LDH assay kit (A020-2; Nanjing Jiancheng Bioengineering). The reaction products were detected by spectrophotometry at 450 nm.

### Measurement of Reactive Oxygen Species and Inflammatory Cytokines

According to the instructions (E004-1-1; Nanjing Jiancheng Bioengineering), the cells were cultured with 2,7-DichlorofuorescinDiacetate (DCFH-DA) probe (10 μm) at 37°C in the dark for 30 min, and cultivated with Gemini EM enzyme (Thermo Fisher Scientific Inc., Fremont, CA, United States) to detect the fluorescence intensity of differently treated cells, which represents the relative ROS content. The levels of TNF-α and IL-1β in differently treated OB-6 cells were recorded according to the above-mentioned experimental methods.

### Flow Cytometry

OB-6 cell apoptosis was detected using an Annexin-V/PI-fluorescein staining kit (Nanjing Keygen Biotech, China) as previously described (Ma et al., 2013). Cells were washed, trypsinized, and centrifuged. Then, cells were collected and re-suspended in a binding buffer for annexin V-FITC and PI staining in the dark for 15 min. The apoptotic cells were counted by flow cytometry (BD Biosciences) and the annexin V/PI ratio reflected the apoptosis percentage. In addition, the caspase-3 activity in OB-6 cells was detected using the above methods.

### Immunofluorescence Assay

Cells were fixed for 30 min with 4% (m/v) paraformaldehyde, washed with PBS, and treated with 0.2% (v/v) Triton X-100 (Solarbio) for 20 min. After three washes with PBS, cells were blocked for 30 min in 5% bovine serum albumin. After that, cells were cultured with primary antibodies against LC3B (1:50, Novus Biological Inc., Littleton, CO, United States) overnight at 4°C. Then samples were added with Cy3-conjugated anti-rabbit secondary antibody (1:300, Servicebio Co., Ltd., Wuhan, Hubei, China) for 1 h. Next, cells were stained with DAPI (Solarbio) for 5 min. Images were obtained by the fluorescence microscopy (Olympus).

### Microarray Analysis

Total RNA was extracted from the femoral head of SANFH rats treated with SANFH and Ato using a TRIzol reagent (Invitrogen). Microarray analysis of miR was completed by Agilent Technology Co., Ltd. (Beijing, China).

### RT-QPCR

TRIzol reagent was used to extract total RNA from OB-6 cells and femoral head tissue homogenates according to the instructions. QuantiTect Reverse Transcription kit (Thermo) was applied to reversely transcribe RNA into cDNA. SYBR Premix Ex Taq II (Takara) was utilized for fluorescence quantitative analysis of cDNA. The primer sequences shown in Table 2 are synthesized by Sangon Biotech Co., Ltd. (Shanghai, China). The relative expression was calculated by the method of 2^−ΔΔCt^ with U6 or β-actin as an internal control.

**TABLE 2:** Primer sequences of RT-qPCR.

Gene	Primer sequence (5′-3′)
rno TLR4	F: GCC​CTG​TTG​GAT​GGA​AAA​GC
	R: ATG​GGT​TTT​AGG​CGC​AGA​GT
rno β-actin	F: ACA​CTC​CAG​CTG​GGC​AAA​GAA​TTC​TCC​TTT
	R: CTC​AAG​TGT​CGT​GGA​GTC​GGC​AA
rno U6	F: CTCGCTTCGGCAGCACA
	R: AAC​GCT​TCA​CGA​ATT​TGC​GT
has U6	F: CTCGCTTCGGCAGCACA
	R: AAC​GCT​TCA​CGA​ATT​TGC​GT
Stem-loop	GTC​GTA​TCC​AGT​GCA​GGG​TCC​GAG​GTA​TTC​GCA​CTG​GAT​ACG​AC
rno miR-30c	F: ATC​GGG​ACC​ATG​TTG​TAG​TGT​G
rno miR-107	F: GCG​GGT​TTC​TCT​CTG​CTT​TAA​G
rno miR-186	F: GAC​GGG​TGC​TTA​CAA​CTT​TCC​A
has miR-186	F: ACG​GGC​TGC​TTG​TAA​CTT​TCC
rno miR-29b	F: AGC​CCG​CTT​CAG​GAA​GCT​GGT​T
rno miR-346	F: CGG​GCT​CTG​TGT​TGG​GCA​TCT
rno miR-26a	F: ATAAGGCCGTGGCCTTGT
rno miR-127	F: CGG​GCT​TTG​ATC​ACT​GTC​TCC
rno miR-122	F: ACG​GGC​CTT​AGC​AGA​GCT​CTG
rno miR-320	F: GCCTCGCTGTCCTCCG
rno miR-34a	F: TGC​CCG​CCG​GCT​GTG​AGT​AAT​T
Universal reverse primer	GTGCAGGGTCCGAGGTAT

Note: RT-qPCR, real-time quantitative polymerase chain reaction; TLR4, toll-like receptor 4; miR, microRNA; F, forward; R, reverse.

### Western Blot Analysis

A standard protein extraction kit (Beyotime, Nanjing, China) was used to extract the total protein in the tissue homogenate and OB-6 cells. Bicinchoninic acid (Nanjing JianCheng Bioengineering) method was employed to determine the protein concentration. Equal volumes of protein samples (30 μg) were loaded on a sodium dodecyl sulfate-polyacrylamide gel (Beyotime, Nanjing, China), and transferred to polyvinylidene fluoride membranes (IPVH00010, Millipore, Bedford, MA, United States). The membranes were probed with primary antibodies against LC3B (1:2,000, ab192890), Beclin-1 (1:1,000, ab210498), TLR4 (1:1,000, ab95562), p38MAPK (1:1,000, ab31828), ERK (1:1,000, ab53277), JNK (1:1,000, ab124956), NF-κB (1:1,000, ab16502), and Tubulin (1:5,000, ab7291) (all from Abcam Inc., Cambridge, MA, United States). Then the membranes were probed with secondary antibody goat anti-rabbit immunoglobulin G (IgG). The protein bands were visualized using Odyssey infrared imaging system (Li-Cor Bioscience, Lincoln, NE, United States) and quantified by Image Pro Plus 6.0 (Media Cybernetics, Silver Spring, United States).

### Statistical Analysis

The sample size of the *in vivo* experiments was six for each group, and the cell experiments were repeated three times. SPSS 19.0 (IBM Corp. Armonk, NY, United States) was employed for data analysis. Kolmogorov-Smirnov test indicated the data were normally distributed. The data are shown as mean ± standard deviation. The *t* test was used for analyzing comparisons between two groups, and one-way or two-way analysis of variance (ANOVA) for comparing different groups, followed by Tukey's multiple comparison test. The *p* value was attained using a two-tailed test and *p* < 0.05 implied a significant difference. GraphPad prism 8.0 (GraphPad software, San Diego, CA, United States) was applied for mapping and calculating the IC50 values.

## Results

### Atorvastatin Relieves SANFH-Induced Damage and Promotes Autophagy

**FIGURE 1:**
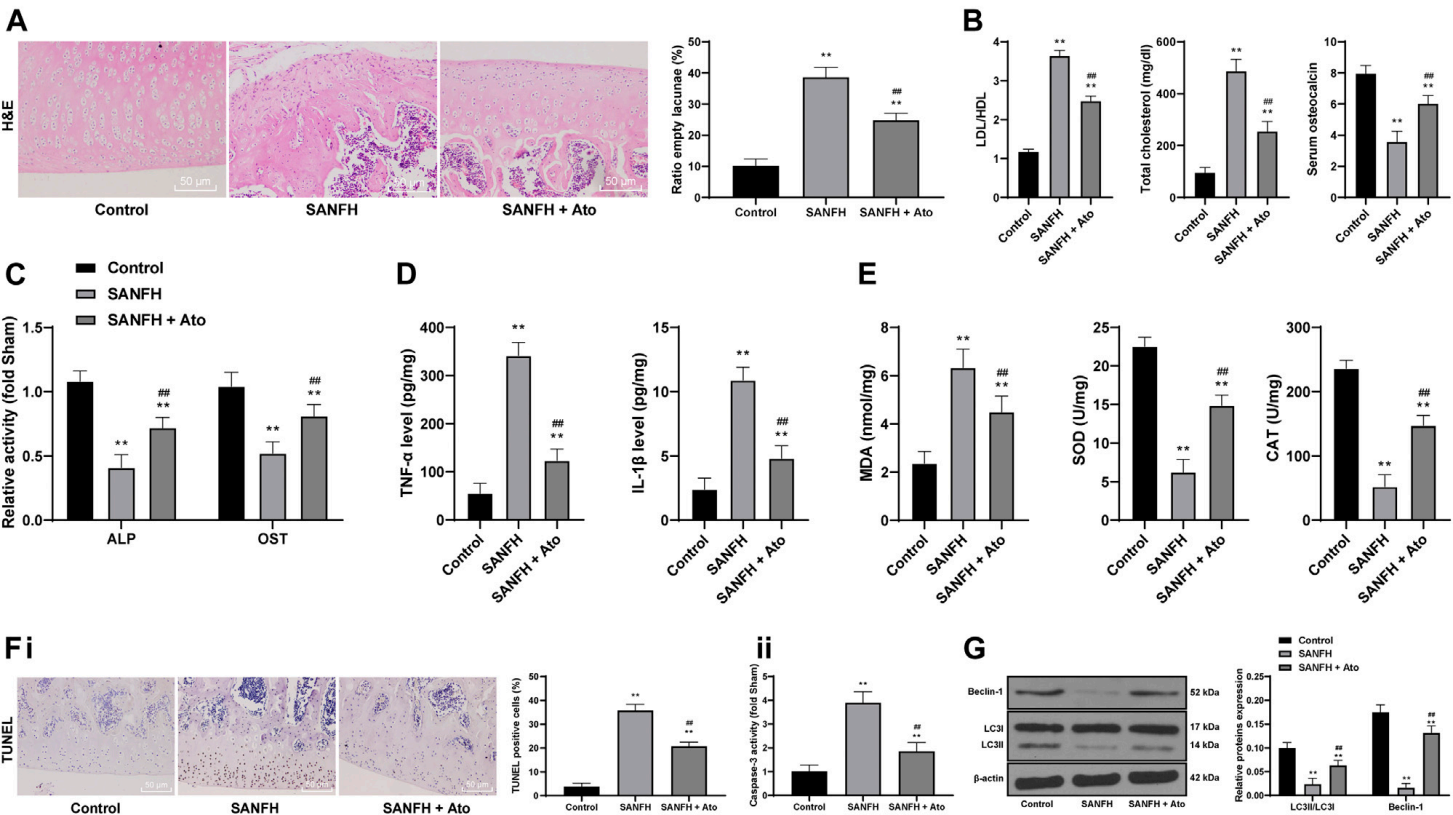
Ato relieves SANFH-induced damage and promotes autophagy. **(A)** HE staining was used to observe the structure of the femoral head and the rate of empty lacunae; the arrows show empty lacunae; **(B)** ELISA measured serum TC, LDL/HDL, and OST content; **(C)** ELISA measured the activity of ALP and OST in the femoral head; **(D)** ELISA measured TNF-α and IL-1β levels in the femoral head; **(E)** ELISA measured MDA, SOD, and CAT levels in the femoral head; **(F)**, apoptosis of the femoral head was detected by TUNEL assay **(i)** and caspase-3 activity test **(ii)**; **(G)** Western blot analysis was used to detect LC3II/LC3I and the expression of Beclin-1 to evaluate the autophagy of rat femoral head. *n* = 6 in each group. The data are shown in mean ± standard deviation. **p < 0.01 vs. control group, ^##^ *p* < 0.01 vs. SANFH group.

HE staining was used to observe the injury of femoral heads in SANFH rats. In control rats, the trabecular bone structure was intact, arranged in order, and the osteocytes were clearly visible, with a normal morphological structure, large nucleus in the center, and a few empty lacunae. In SANFH rats, the trabeculae were sparse, thin, disordered, broken, and fragmented, showing typical osteonecrosis, and empty lacunae was increased significantly. After oral administration of Ato, the trabecular structure of rats in the SANFH group was basically complete but arranged in a disordered way. Compared with SANFH rats, the rate of empty lacunae was significantly reduced (Figure 1A, all *p* < 0.01).

Compared with the control rats, SANFH rats had higher serum TC and LDL/HDL, and lower serum OST content (the bone metabolism markers), while the TC and LDL/HDL of the SANFH rats treated with Ato were lower, and the OST content was higher (Figure 1B, all *p* < 0.01). In addition, compared with control rats, the activities of ALP and OST, TNF-α and IL-1β, MDA, SOD, and CAT were significantly decreased in SANFH rats. The activity of osteoblasts, inflammatory responses, and oxidative stress were significantly increased after Ato administration (Figure 1C) (all *p* < 0.01).

Additionally, we evaluated apoptosis by TUNEL assay and caspase-3 activity test. Compared with those in control rats, TUNEL-positive cells and caspase-3 activity in the femoral head of SANFH rats were increased, while the indexes related to apoptosis were decreased significantly after Ato treatment (Figure 1F), (all *p* < 0.01). Western blot analysis evaluated LC3II/LC3I and Beclin-1 expression to evaluate the autophagy of rat femoral heads. Compared with control rats, SANFH rats showed reduced LC3II/LC3I and Beclin-1 expression, indicating that autophagy was inhibited. Compared with that in SANFH rats, LC3II/LC3I and Beclin-1 expression in SANFH rats treated with Ato were significantly increased (Figure 1G, *p* < 0.01).

### miR-186 is Upregulated in Atorvastatin-Treated SANFH Rats

**FIGURE 2:**
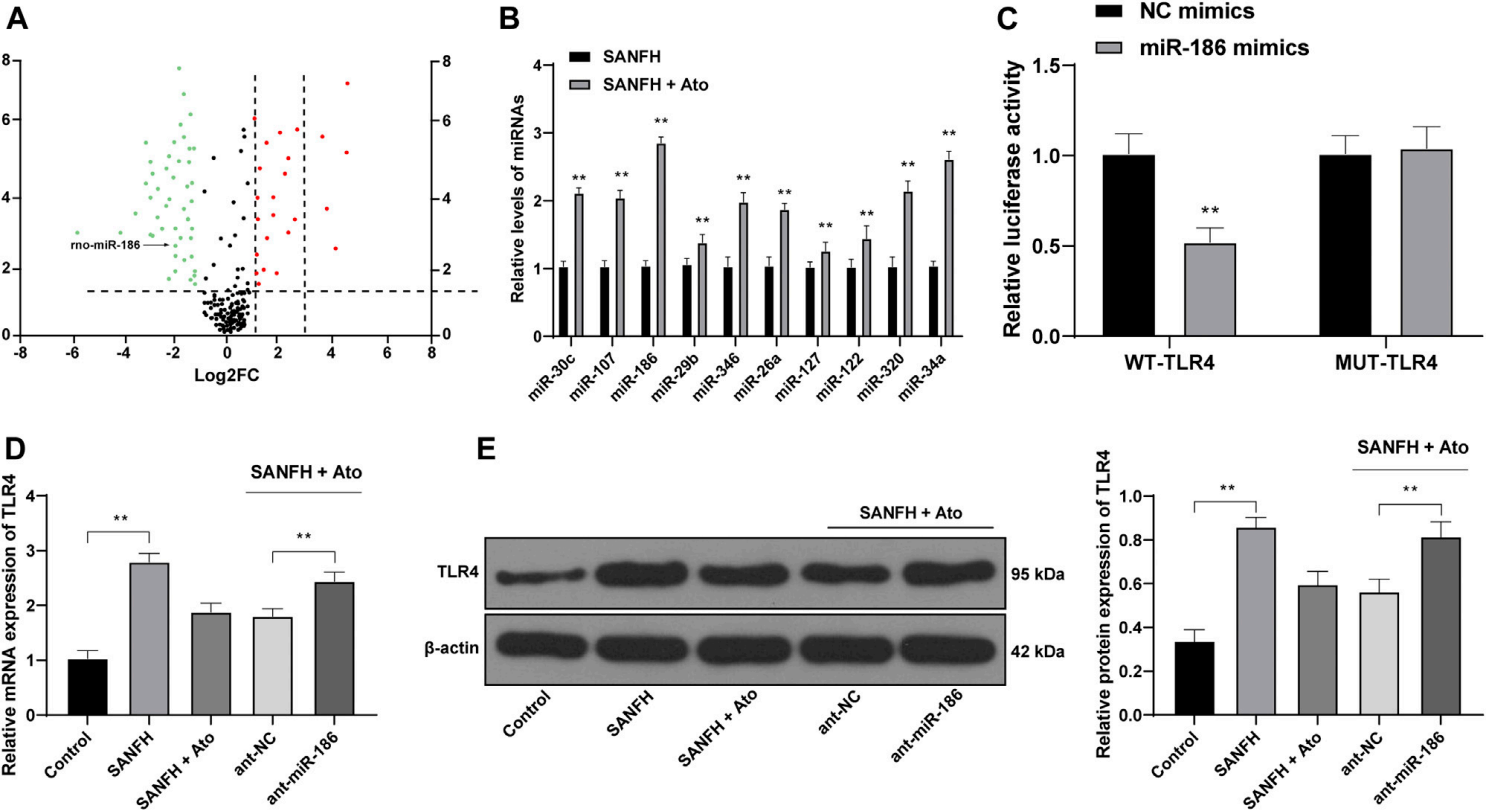
miR-186 is upregulated in Ato-treated SANFH rats and targets TLR4. **(A)** The differentially expressed miRs in the femoral head of rats in the SANFH + Ato group were analyzed by microarray; each point represents a miR; green represents the downregulated miR in the femoral head of SANFH rats after Ato treatment, and red represents the upregulated miR; **(B)** 10 miRs with high expression in SANFH + Ato rats were selected and further verified by RT-qPCR; **(C)** bioinformatics predicted there was a specific binding site between miR-186 and TLR4, and the target binding relationship was proven by the dual luciferase reporter gene assay; **(D,E)** levels of TLR4 mRNA and protein after inhibiting miR-186 expression were detected by RT-qPCR and Western blot analysis. n = 6 in each group. The data are shown in mean ± standard deviation. **p < 0.01 vs. control group, or SANFH group.

To explore the protective effect of Ato on SANFH rats, we used miR in the femoral head of rats as the entry point. The differentially expressed miRs in the femoral heads of rats in SANFH + Ato group were analyzed by microarray and screened with |Log2FC|>1 and *p* < 0.05 as the screening criteria. In SANFH rats treated with Ato, we screened 72 miRs with differential expression, 24 of which were upregulated and 48 were downregulated (Figure 2A). Ten miRs with high expression in SANFH + Ato rats were selected and further verified by RT-qPCR. miR-186 was the most significantly increased (Figure 2B, *p* < 0.01). According to the prediction of bioinformatics, there was a specific binding site between miR-186 and TLR4, and the target binding relationship was proven by the dual luciferase report assay (Figure 2C, *p* < 0.01). Then, by inhibiting the expression of miR-186 in the femoral heads of SANFH rats after ATO treatment, TLR4 levels in the ant-miR-186 group were higher than those in the ant-NC group (Figures 2D,E, both *p* < 0.01).

### miR-186 Inhibits TLR4 to Alleviate SANFH-Induced Injury After Atorvastatin Treatment

**FIGURE 3:**
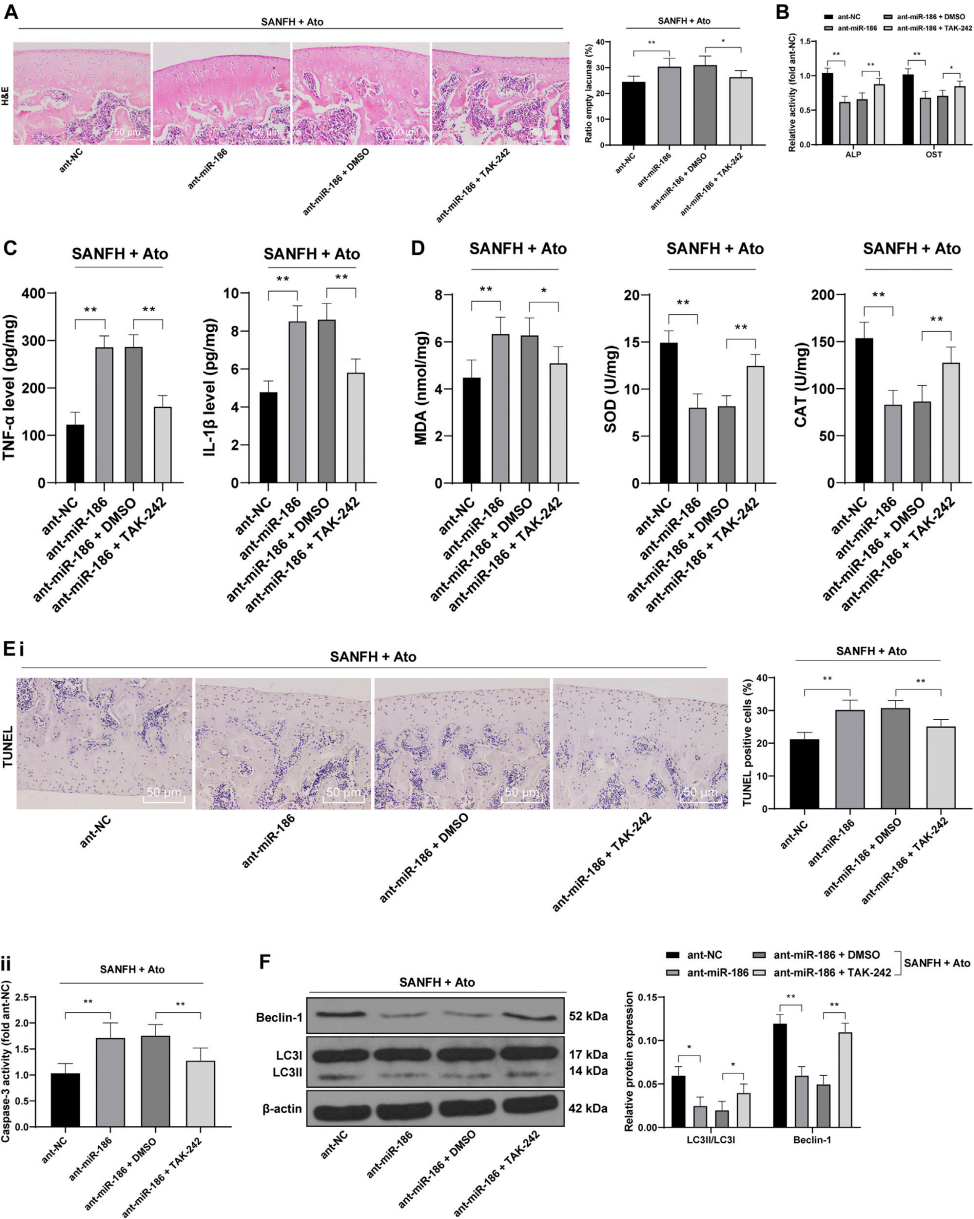
miR-186 inhibits TLR4 to alleviate SANFH-induced injury after Ato treatment. After Ato treatment, SANFH rats were injected with miR-186 antagomir or TAK242. **(A)** HE staining was used to observe the structure of the femoral head and the rate of empty lacunae; **(B)** ELISA measured the activity of ALP and OST in the femoral head; **(C)** ELISA measured TNF-α and IL-1β levels in the femoral head; **(D)** ELISA measured MDA, SOD, and CAT levels in the femoral head; **(E)** apoptosis of the femoral head was detected by TUNEL assay **(i)** and caspase-3 activity test **(ii)**; **(F)** Western blot analysis was used to detect LC3II/LC3I and the expression of Beclin-1 to evaluate the autophagy of rat femoral head. *n* = 6 in each group. The data are shown in mean ± standard deviation. *p < 0.05, **p < 0.01.

After Ato treatment, SANFH rats were injected with miR-186 antagomir or TAK242, a TLR4 inhibitor, to observe whether miR-186 regulates TLR4 and affects the femoral head injury and autophagy of SANFH rats. HE staining was used to observe the histopathological changes of the femoral heads in rats. Compared with those in the ant-NC group, the structure of the femoral heads in the ant-miR-186 group was disordered and the rate of empty lacunae was significantly increased, showing the characteristics of osteonecrosis. TAJK242 reversed the damage of the femoral heads caused by ant-miR-186, and the rate of empty lacunae was significantly reduced (Figure 3A, *p* < 0.01). In addition, compared with those in ant-NC-operated rats, the activities of ALP and OST, TNF-α and IL-1β, and MDA were significantly decreased in ant-miR-186-treated rats, while SOD and CAT were downregulated. The treatment of ant-miR-186 + TAK242 reversed these outcomes in ant-miR-186-treated rats (Figures 3B–D) (all *p* < 0.01).

Compared with those in ant-NC-operated rats, TUNEL-positive cells and caspase-3 activity in the femoral heads of ant-miR-186-treated rats were increased, while the indexes related to apoptosis were decreased significantly after treatment with ant-miR-186 + TAK242 (Figure 3E, all *p* < 0.01). In the ant-miR-186 group, LC3II/LC3I and Beclin-1 expression were significantly downregulated, and autophagy was reduced, while TAK242 was able to restore the autophagy level of rats to a certain extent (Figure 3F, all *p* < 0.01). All the above results indicated that miR-186 targets TLR4 in SANFH rats and thus inhibits SANFH-induced injury.

### Atorvastatin Inhibits the Activation of the TLR4/MAPKs/NF-κB Pathway via Upregulation of miR-186

**FIGURE 4:**
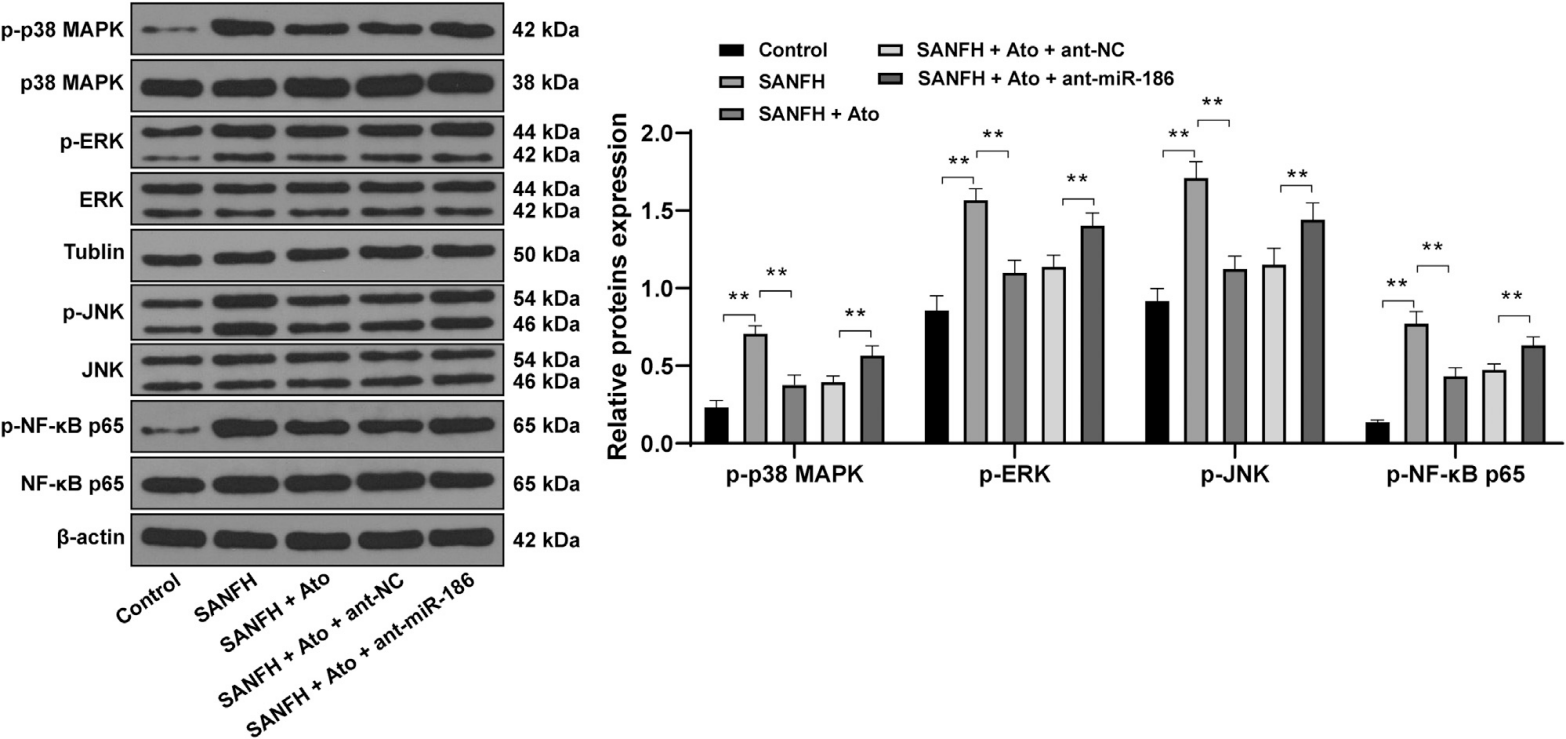
Ato inhibits the activation of the MAPKs/NF-κB pathway in SANFH. The expression of MAPK/NF-κB pathway-related proteins by Western blot. *n* = 6 in each group. **p < 0.01.

Then our focus shifted to the downstream pathway involved in the protective effects of Ato on SANFH rats. Compared with control rats, phosphorylation of NF-κB p65 and upstream regulatory proteins of the NF-κB MAPK family (p38 MAPK, ERK, and JNK) in the femoral heads of SANFH rats were significantly increased. Compared with SANFH rats, Ato-treated rats had significantly decreased phosphorylation of p38 MAPK, ERK, JNK, and NF-κB. In addition, when we inhibited the expression of miR-186 in SANFH rats treated with Ato, compared with those in the SANFH + Ato + ant-NC group, MAPK/NF-κB pathway-related proteins in SANFH + Ato + ant-miR-186 group were all significantly increased (Figure 4, all *p* < 0.01).

### Atorvastatin Reduces DEX-Induced Osteoblast Injury

**FIGURE 5:**
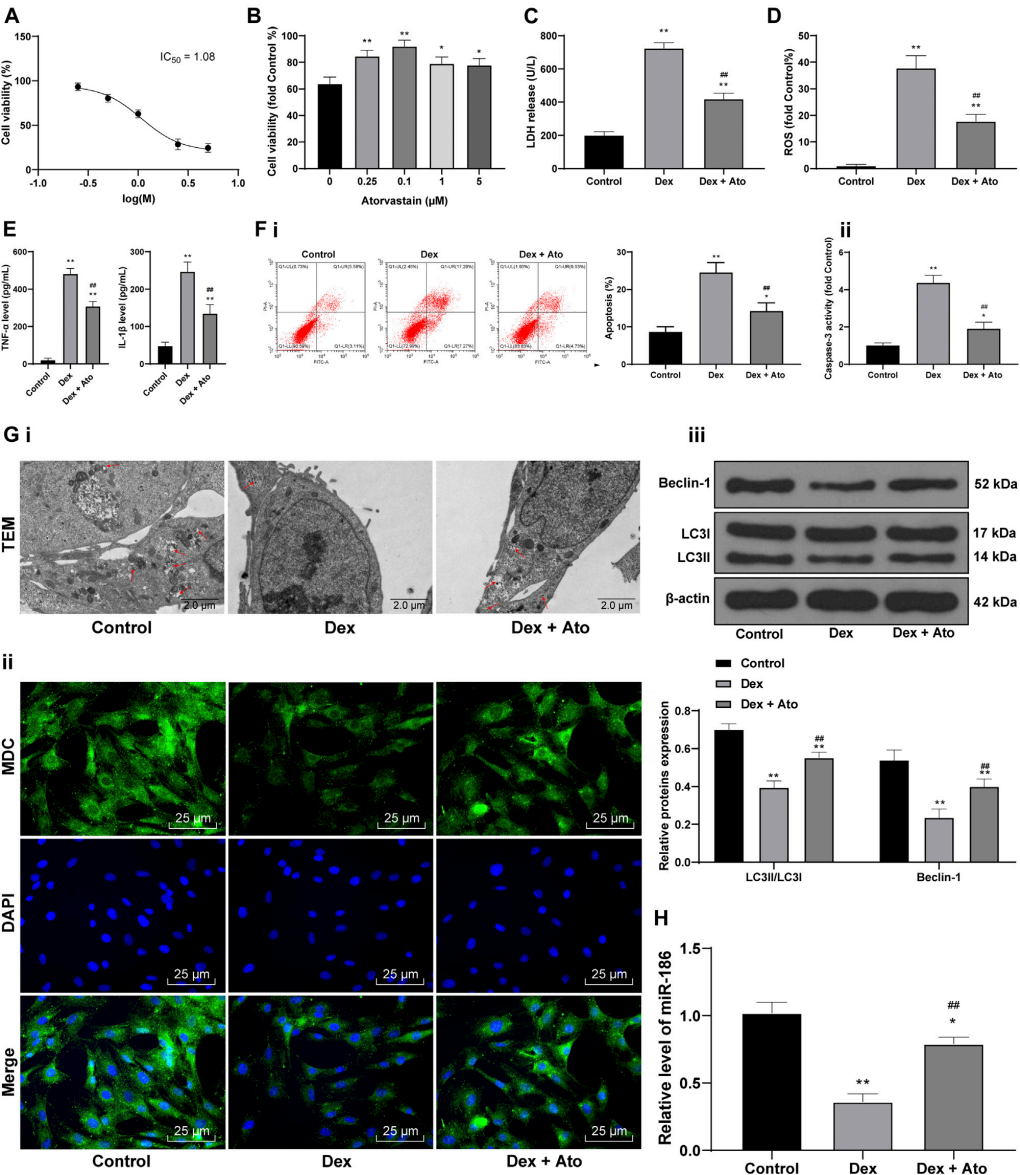
Ato reduces DEX-induced osteoblast injury. **(A)** MTT assay detected OB-6 cell viability at the concentration of DEX; **(B)** MTT assay detected 1 μM DEX-treated OB-6 cell viability after treatment with different concentrations of Ato for 72 h; **(C)** Relative LDH release in OB-6 cells was analyzed by LDH assay kit; **(D)** ROS level in OB-6 cells was measured by DCFH-DA probe; **(E)** levels of inflammatory cytokines (TNF-α and IL-1β) were detected by ELISA kits; **(F)** apoptosis of femoral head was detected by flow cytometry **(i)** and caspase-3 activity test **(ii)**; **(G)** transmission electron microscopy **(i)** and MDC fluorescence staining **(ii)** were used to observe autophagy; **(iii)** Western blot analysis was used to detect LC3II/LC3I and Beclin-1 expression; **(H)** RT-qPCR detected miR-186 expression in osteoblasts. Replicates = 3. The data are shown in mean ± standard deviation. *p < 0.05, **p < 0.01, vs. the control group; ^#^ *p* < 0.05, ^##^ *p* < 0.01 vs. DEX group.

To further explore the effect of Ato on SANFH, 0, 0.25, 0.5, 1, 2.5, and 5 μM DEX were set to pretreat OB-6 osteoblasts for 24 h, and then the cell survival rate was determined. Then we calculated and analyzed the cell survival rate of different concentrations of DEX-treated cells, and calculated the IC50 of DEX as 1.08. According to the method in the references (Huang et al., 2019b), 1 μM DEX-treated OB-6 cells were selected to simulate SANFH *in vitro* model for subsequent experiments (Figure 5A). After OB-6 cells were pretreated with DEX and treated with 0, 0.25, 0.1, 1, and 5 μM Ato respectively, the cells treated with 0.1 μM Ato had the highest survival rate (Figure 5B, *p* < 0.05). Therefore, 0.1 μM Ato was selected for follow-up experiments. In the DEX group, LDH release, ROS, and inflammatory cytokines were enhanced, while apoptosis was decreased vs. those in the control group; in the DEX + Ato group, LDH, ROS levels, and levels of inflammatory cytokines were significantly reduced and apoptosis was enhanced compared with those in the DEX group (Figures 5C–F, all *p* < 0.05). Transmission electron microscopy and MDC staining were used to observe autophagy, and the expression of LC3II/LC3I and Beclin-1 was detected by Western blot analysis. The autophagosomes decreased in the DEX group, the fluorescence intensity of MDC and the punctate structure was decreased, and the LC3II/LC3I and Beclin-1 were significantly reduced. The autophagy-related indexes in cells treated with Ato were recovered to a certain extent (Figure 5G, all *p* < 0.05). RT-qPCR showed that miR-186 expression in osteoblasts decreased after DEX treatment, but increased after Ato treatment (Figure 5H, all *p* < 0.05).

### Atorvastatin Relieves DEX-Induced Osteoblast Injury via Upregulation of miR-186

**FIGURE 6:**
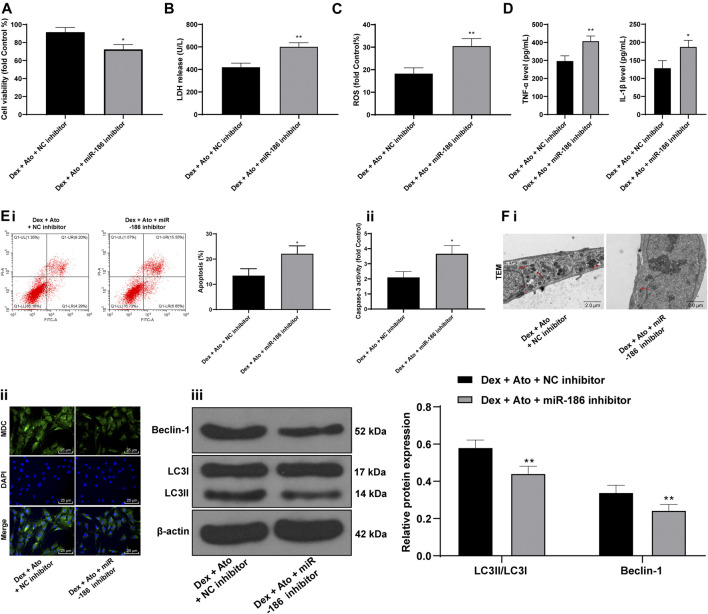
Ato relieves DEX-induced osteoblast injury via upregulation of miR-186. DEX-pretreated OB-6 cells were selected to simulate the *in vitro* model of SANFH, then treated with 0.1 μM Ato and transfected with miR-186 inhibitor, and the cell damage and autophagy were observed. **(A)** MTT assay detected OB-6 cell viability; **(B)** Relative LDH release in OB-6 cells was analyzed by an LDH assay kit; **(C)** ROS level in OB-6 cells was measured by DCFH-DA probe; **(D)** levels of inflammatory cytokines (TNF-α and IL-1β) were detected by ELISA kits; **(E)** apoptosis of femoral head was detected by flow cytometry **(i)** and caspase-3 activity test **(ii)**; **(F)** transmission electron microscopy **(i)** and MDC fluorescence staining **(ii)** were used to observe autophagy; **(iii)**, western blot analysis was used to detect LC3II/LC3I and Beclin-1 expression. Replicates = 3. The data are shown in mean ± standard deviation. *p < 0.05, **p < 0.01, vs. the control group; ^#^ *p* < 0.05, ^##^ *p* < 0.01 vs. DEX group.

DEX-pretreated OB-6 cells were selected to simulate the *in vitro* model of SANFH, then treated with 0.1 μM Ato and transfected with miR-186 inhibitor to observe the cell damage and autophagy. Relative to the DEX + Ato + NC inhibitor group, the DEX + Ato + miR-186 inhibitor group showed a decrease in cell viability (Figure 6A) and increases in LDH release (Figure 6B), ROS content (Figure 6C), inflammatory cytokines (Figure 6D), and apoptosis (Figure 6E, all *p* < 0.01); it reduced LC3II/LC3I and Beclin-1, autophagosomes, and fluorescence intensities of MDC (Figure 6F, all *p* < 0.05).

### Atorvastatin Upregulates miR-186 to Inactivate the TLR4/MAPKs/NF-κB Axis in DEX-Induced OB-6 Osteoblasts

**FIGURE 7:**
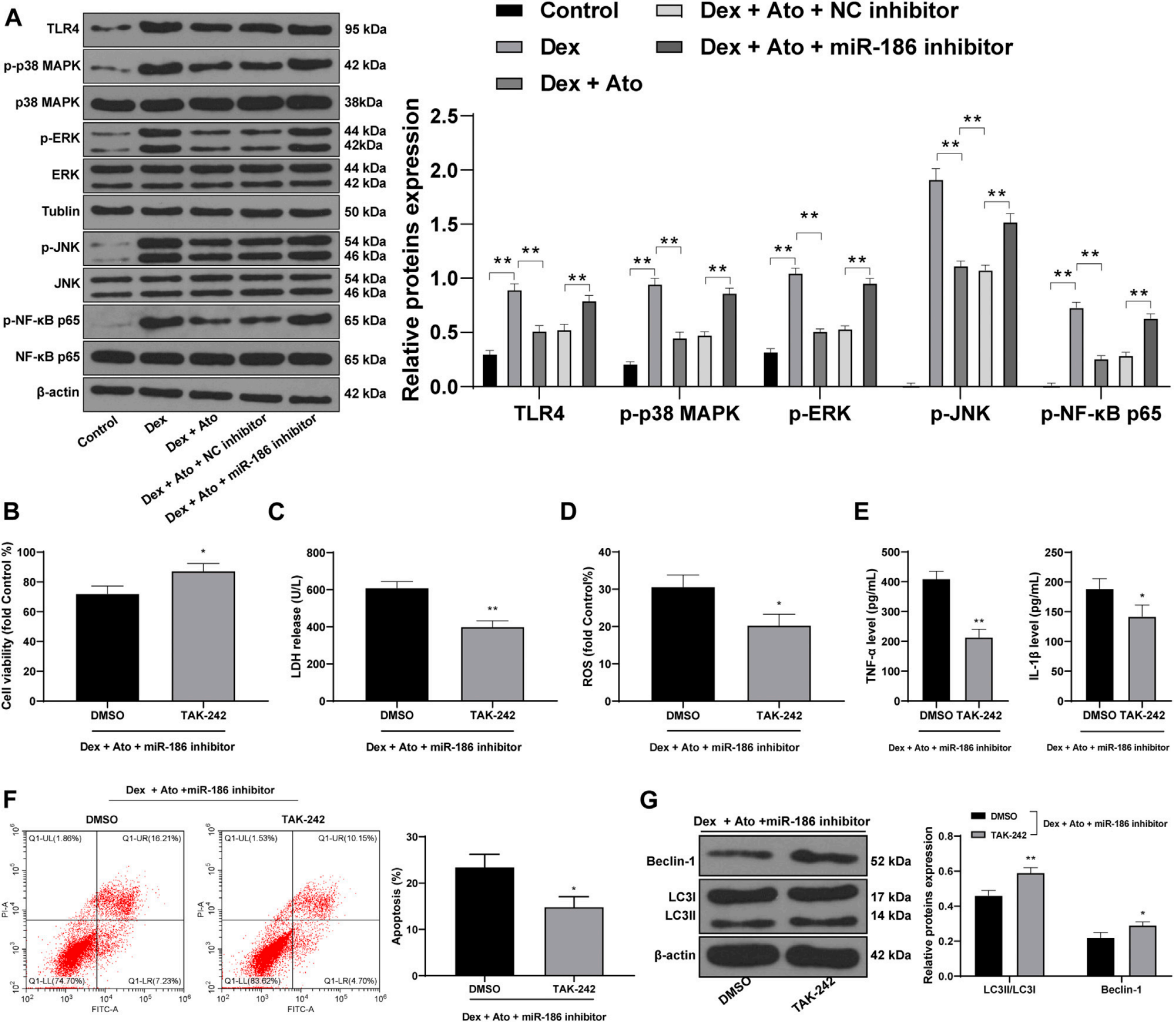
Ato upregulates miR-186 to inactivate the TLR4/MAPKs/NF-κB axis in DEX-induced OB-6 osteoblasts. **(A)** the expression of MAPK/NF-κB pathway-related proteins detected by Western blot; **(B)** MTT assay detected OB-6 cell viability; **(C)** Relative LDH release in OB-6 cells was analyzed by LDH assay kit; **(D)** ROS level in OB-6 cells was measured by DCFH-DA probe; **(E)** levels of inflammatory cytokines (TNF-α and IL-1β) were detected by ELISA kits; **(F)** apoptosis of femoral head was detected by flow cytometry; **(G)** Western blot analysis was used to detect LC3II/LC3I and Beclin-1 expression. Replicates = 3. The data are shown in mean ± standard deviation. *p < 0.05, **p < 0.01, vs. the DEX + Ato + NC inhibitor + DMSO group.

We further detected the activation of the TLR4/MAPKs/NF-κB pathway in OB-6 cells of each group. Western blot analysis showed that TLR4 level in DEX-pretreated cells and phosphorylation of p38 MAPK, ERK, JNK, and NF-κB p65 were markedly increased, but after the action of Ato, the levels of these proteins were downregulated. Further inhibition of miR-186 partially reversed the inhibitory effects of Ato on the TLR4/MAPK/NF-κB pathway-related protein expression (Figure 7A, all *p* < 0.01).

TAK242, a TLR4 inhibitor, was used to treat cells in the DEX + ATO + miR-186 inhibitor group, and the cell damage was detected. Compared with cells in the DEX + ATO + miR-186 inhibitor + DMSO group, OB-6 cells in the DEX + ATO + miR-186 inhibitor + TAK242 group had increased viability, decreased LDH release, ROS, TNF-α, and IL-1 β (Figures 7B–E, all *p* < 0.01), reduced apoptosis, and enhanced autophagy level (Figures 7F,G, all *p* < 0.01).

## Discussion

SANFH is one of the most common hip pain diseases, and its incidence has been on the rise in recent years (Erken et al., 2012; Li et al., 2019). Although numerous therapeutic procedures have been implemented to treat SANFH, most of them failed to show satisfactory clinical outcomes, especially for young patients who have developed SANFH (Erken et al., 2012). It has been documented that a lipid-lowering agent (lovastatin) could prevent the occurrence of steroid-induced osteonecrosis in rabbits (Kang et al., 2010). Li et al. found a subset of miRs that were differentially expressed by more than two-fold in the collapse area compared to the non-collapse area in three patients with SANFH (Li et al., 2017). Therefore, we postulate that another lipid-lowering agent (Ato) can prevent the conditions associated with the development of SNAFH through miR regulation. To answer the question, the effect of Ato on the prevention of SNAFH was evaluated in the present study. The results demonstrated that Ato downregulated apoptosis and promoted autophagy, thus alleviating SANFH via miR-186 and the TLR4-mediated MAPKs/NF-κB pathway.

Increased blood lipids cause a serious fat embolism condition, in which adipose cells occupy bone marrow cells and then fuse into pieces and induce the death of marrow-derived cells, resulting in SANFH (Li et al., 2019). In this study, after oral administration of Ato, the TC and LDL/HDL of SANFH rats were reduced, and the activity of osteoblasts, inflammatory responses, and oxidative stress were notably elevated. A plethora of primary and secondary prevention studies of statins have shown conclusively that lowering LDL-C, TC, and TG levels or raising HDL-C levels reduces cardiovascular events (Ai et al., 2018). Ato was also found to inhibit ALT and AST activities in rats with streptozotocin-induced diabetes, and significantly reduced lipid peroxidation and oxidative damage (Aktay et al., 2019). Ato administration alleviates oxidative stress, preserves SOD levels, and decreases MDA levels (Zhang et al., 2017). Ato treatment attenuated renal lipid accumulation-induced lipotoxicity in high-fat diet-fed rats, and the proteins involved in renal inflammation, fibrosis, oxidative stress, and apoptosis were attenuated by Ato treatment (Pengrattanachot et al., 2020). Elevated caspase-3 expression is frequently observed in SANFH patients (Xu et al., 2014). The apoptosis analyses in this study indicated that TUNEL-positive cells and caspase-3 activity in the femoral head tissue of model rats were dramatically elevated, but decreased after Ato treatment. Ato visibly decreased caspase-3 expression in neurons, astrocytes, and oligodendrocytes and limited inflammatory responses after a spinal cord injury (Bimbova et al., 2018). These results indicated that Ato can reduce the injury of SANFH rats by inhibiting inflammation, oxidative stress, and apoptosis, and promoting autophagy.

After knowing the protective effects of Ato on SANFH, we tried to figure out the deep molecular mechanism. miR microarray performed in a prior study identified the differentially abundant miRs and supported that circulating miRs may serve as diagnostic markers and therapeutic targets for SANFH (Li et al., 2018). In this study, through microarray analysis and expression detection, we verified that miR-186 was upregulated in SANFH rats after Ato treatment. miR-186 was reported to activate the bone morphogenetic protein pathway to promote fracture healing in a mouse model of femoral fracture (Wang et al., 2019a). In this study, miR-186 inhibition reversed the protection of Ato on SANFH rats. miR-186–5p upregulation prevents viability and promotes autophagy in hepatocellular carcinoma (Shan and Li, 2019). Our study made a novel investigation of miR-186 in SANFH. In addition, miR-186 was verified to target TLR4. TLR4 participates in the regulation of miR-186 in the anti-apoptotic effect on podocytes (Sha et al., 2015). The TLR4 pathway is pivotal in the progression of steroid-induced osteonecrosis (Tian et al., 2014). TLR4 stimulation and corticosteroid interactively induce osteonecrosis of the femoral head in rats (Okazaki et al., 2016). Moreover, our results discovered that inhibition of TLR4 expression restored the protective effect of Ato on SANFH rats. SANFH may be caused by disruption of the immune system via lipopolysaccharide-activated TLR4 signaling and abnormal lipid synthesis or metabolism (Okazaki et al., 2009). The inhibition of TLR4 expression improves bone metabolism, promotes ALP and bone mineralization, and reduces hyperglycemia-induced osteoblast apoptosis, levels of inflammatory factors, and the expression of Beclin 1 and LC3II/LC-I (Zhang et al., 2020).

Furthermore, we shifted to exploring the downstream pathway of TLR4. TLR4/MAPKs are found to regulate the expression of NF-κB and autophagy (Zhou et al., 2018). As expected, Ato upregulated miR-186 to inactivate the TLR4/MAPKs/NF-κB axis in SNAFH rats and DEX-induced OB-6 osteoblasts. TLR4 activates the MyD88 pathway that stimulates NF-κB, JNK, and ERK and induces inflammatory cytokine expression (Rui et al., 2006). The excessive activation of the TLR4/NF-κB pathway is closely associated with rat femur necrosis, and its activator lipopolysaccharide can notably increase the incidence rate of SANFH (Wu et al., 2008; Pei et al., 2017). In an *in vitro* SNAFH model using Murine preosteoblast MC3T3-E1 cells, MAPKs are closely related to ROS-induced osteoblast apoptosis in a high-dose DEX environment (Bai et al., 2019). Ato treatment significantly increases the level of SOD, downregulates MDA, ROS, and LDH, inhibits levels of TLR4, NF-κB, NLRP3 and cleaved caspase-1, and blocks the secretion of inflammatory cytokines in HK-2 cells and rat kidney tissue (Sun et al., 2020). Osteoblasts are the main target of glucocorticoid-induced apoptosis for the model of femoral head osteonecrosis (Feng et al., 2017). The results *in vitro* further verified that Ato reduced the apoptosis and promoted autophagy of OB-6 osteoblasts by upregulating miR-186 and blocking the TLR4/MAPKs/NF-κB pathway.

In conclusion, our experimental results supported that Ato downregulated apoptosis and promoted autophagy to alleviate SANFH via miR-186 and TLR4-mediated MAPKs/NF-κB pathway. These results provide a potential mechanism and potentially refine our understanding of SANFH. Further clinical studies exploring the effects of Ato and miR-186 on mediating SANFH will likely increase our knowledge of the application value.

## Data Availability

The raw data supporting the conclusion of this article will be made available by the authors, without undue reservation.
