# Supplementary material for: Atorvastatin Upregulates microRNA-186 and Inhibits the TLR4-Mediated MAPKs/NF-κB Pathway to Relieve Steroid-Induced Avascular Necrosis of the Femoral Head
Source: Front Pharmacol. 2021 Apr 16;12:583975. doi: 10.3389/fphar.2021.583975 (PMC8115218; doi:10.3389/fphar.2021.583975)
Supplement: Supplementary file 1 [file Image1.tiff]

Frontiers | Atorvastatin upregulates microRNA-186 and inhibits the TLR4-mediated MAPKs/NF-κB pathway to relieve steroid-induced avascular necrosis of the femoral head | Pharmacology


Login
 / 
Register

- About
- Journals
- Research Topics
- Articles
- More

Submit

Login
 / 
Register

Submit

**Impact Factor 4.225** | **CiteScore 5.0**More on impact ›

|  |  |
| --- | --- |
| Frontiers in Pharmacology | Inflammation Pharmacology |

Toggle navigation


Section


- (current)Section
- About
- Articles
- Research topics
- For authors 
  - Why submit?
  - Fees
  - Article types
  - Author guidelines
  - Review guidelines
  - Submission checklist
  - Contact editorial office
  - Submit your manuscript
- Editorial board

- *Article alerts*

Articles


**Suggest a Research Topic >**

- 53
  total views

 View Article Impact

**Suggest a Research Topic >**

##### SHARE ON

- Facebook

  0
- Twitter

  0
- LinkedIn

  0
- AddThis

  New


## Original Research ARTICLE

Front. Pharmacol.
| doi: 10.3389/fphar.2021.583975

# Atorvastatin upregulates microRNA-186 and inhibits the TLR4-mediated MAPKs/NF-κB pathway to relieve steroid-induced avascular necrosis of the femoral head Provisionally accepted The final, formatted version of the article will be published soon. **Notify me**

Wenhua Huang1\*, Yusong Zhang1, 
Limin Ma1 and 
Erhai Lu1

- 1Southern Medical University, China

Steroid-induced avascular necrosis of the femoral head (SANFH) is caused by the death of active components of femoral head owing to over doses of hormones. The use of lipid-lowering drugs to prevent SANFH in animals inspired us to identify the mechanisms involving Atorvastatin (Ato) in SANFH. However, it is still not well understood about how and to what extent Ato affects SANFH. This study aimed to figure out the efficacy of Ato in SANFH and the underlying molecular mechanisms. After establishment of SANFH model, histological evaluation, lipid metabolism, inflammatory cytokines, oxidative stress, apoptosis and autophagy of femoral head were evaluated. The differentially expressed microRNAs (miRs) after Ato treatment were screened out using microarray analysis. The downstream gene and pathway of miR-186 were predicted and their involvement in SANFH rats was analyzed. OB-6 cells were selected to simulate SANFH in vitro. Cell viability, cell damage, inflammation responses, apoptosis, and autophagy were assessed. Ato alleviated SANFH, inhibited apoptosis and promoted autophagy. miR-186 was significantly upregulated after Ato treatment. miR-186 targeted TLR4 and inactivated the MAPKs/NF-κB pathway. Inhibition of miR-186 reversed the protection of Ato on SANFH rats, while inhibition of TLR4 restored the protective effect of Ato. Ato reduced apoptosis and promoted autophagy of OB-6 cells by upregulating miR-186 and inhibiting the TLR4/MAPKs/NF-κB pathway. In conclusion, Ato reduced apoptosis and promoted autophagy, thus alleviating SANFH via miR-186 and TLR4-mediated MAPKs/NF-κB pathway.

Keywords: 
Steroid induced avascular necrosis of the femoral head, Autophagy, atorvastatin, microRNA-186, TLR4, MAPKs/NF-κB pathway

Received: 17 Jul 2020;
Accepted: 03 Feb 2021.

Copyright: © 2021 Huang, Zhang, Ma and Lu. This is an open-access article distributed under the terms of the Creative Commons Attribution License (CC BY). The use, distribution or reproduction in other forums is permitted, provided the original author(s) and the copyright owner(s) are credited and that the original publication in this journal is cited, in accordance with accepted academic practice. No use, distribution or reproduction is permitted which does not comply with these terms.

\* Correspondence: 
PhD. Wenhua Huang, Southern Medical University, Guangzhou, China, drhuangwh1029@163.com

Write a comment...

Add

##### COMMENTARY

##### ORIGINAL ARTICLE

##### People also looked at

**Suggest a Research Topic >**

×

#### Supplementary Material

  

There is no supplementary material currently available for this article

Loading supplemental data...

  

|  | File Name |  |
| --- | --- | --- |
|  | Table 1.DOCX |  |
|  | Table 2.DOCX |  |
|  | Image 1.TIFF |  |

  

Close

- About Frontiers
- Institutional Membership
- Books
- News
- Frontiers' social media
- Contact
- Careers
- Submit
- Newsletter
- Help Center
- Terms & Conditions
- Privacy Policy

© 2007 - 2021 Frontiers Media S.A. All Rights Reserved

### Privacy Preference Center

Our website uses cookies that are necessary for its operation. Additional cookies are only used with your consent. These cookies are used to store and access information such as the characteristics of your device as well as certain personal data (IP address, navigation usage, geolocation data) and we process them to analyse the traffic on our website in order to provide you a better user experience, evaluate the efficiency of our communications and to personalise content to your interests. Some cookies are placed by third-party companies with which we work to deliver relevant ads on social media and the internet. Click on the different categories' headings to change your cookie preferences. Click on "More Information" if you wish to learn more about how data is collected and shared.
More information

### Manage Consent Preferences

#### Strictly Necessary Cookies

Always Active

These cookies are necessary for the website to function and cannot be switched off in our systems. They are usually only set in response to actions made by you which amount to a request for services, such as setting your privacy preferences, logging in or filling in forms. You can set your browser to block or alert you about these cookies, but some parts of the site will not then work. These cookies do not store any personally identifiable information.

#### Analytics Cookies

Analytics Cookies

These cookies allow us to count visits and traffic sources so we can measure and improve the performance of our site. They help us analyse which pages are the most and least popular and see how visitors move around the site.    All information these cookies collect is aggregated and therefore anonymous.

#### Functional Cookies

Functional Cookies

These cookies enable the website to provide enhanced functionality and personalisation. They may be set by us or by third party providers whose services we have added to our pages. If you do not allow these cookies then some or all of these services may not function properly.

#### Advertising Cookies

Advertising Cookies

These cookies may be set through our site by our advertising partners. They may be used by those companies to build a profile of your interests and show you relevant adverts on other sites.    They do not store directly personal information, but are based on uniquely identifying your browser and internet device. If you do not allow these cookies, you will experience less targeted advertising.

### Back Button Performance Cookies

Vendor Search  Search Icon

Filter Icon

Clear

checkbox label label

Apply Cancel

Consent Leg.Interest

checkbox label label

checkbox label label

checkbox label label

Confirm My Choices
